# Highly Pathogenic Avian Influenza A(H5N1) Virus Clade 2.3.4.4b in Wild Birds and Live Bird Markets, Egypt

**DOI:** 10.3390/pathogens12010036

**Published:** 2022-12-26

**Authors:** Rabeh El-Shesheny, Yassmin Moatasim, Sara H. Mahmoud, Yi Song, Ahmed El Taweel, Mokhtar Gomaa, Mina Nabil Kamel, Mohamed El Sayes, Ahmed Kandeil, Tommy T. Y. Lam, Pamela P. McKenzie, Richard J. Webby, Ghazi Kayali, Mohamed Ahmed Ali

**Affiliations:** 1 Center of Scientific Excellence for Influenza Viruses, National Research Centre, Giza 12622, Egypt; 2 State Key Laboratory of Emerging Infectious Diseases, School of Public Health, The University of Hong Kong, Hong Kong SAR, China; 3 Laboratory of Data Discovery for Health Limited, Hong Kong SAR, China; 4 Department of Infectious Diseases, St. Jude Children’s Research Hospital, Memphis, TN 38105, USA; 5 Centre for Immunology & Infection Limited, Hong Kong SAR, China; 6 Human Link, Dubai 3O-01-BA380, United Arab Emirates

**Keywords:** Egypt, H5N1, live bird markets, poultry, wild birds

## Abstract

Clade 2.3.4.4 H5Nx influenza viruses have further diversified into several subclades. Sub-clade 2.3.4.4b H5N1 viruses have been widely circulating in wild birds and detected in Europe, Africa, Asia, and North America since October 2020. In this study, we report the first detection of highly pathogenic avian influenza H5N1 clade 2.3.4.4b viruses in wild birds and domestic ducks from live bird markets in Egypt. Phylogenetic analysis revealed that the Egyptian H5N1 virus retained the genomic composition of Eurasian strains. Mutations in the viral proteins associated with zoonotic potential and pathogenicity were detected in Egyptian isolates. Egypt is considered a hot spot for the evolution of the influenza virus, so active surveillance of avian influenza viruses in Egypt is warranted.

## 1. Introduction

Infectious diseases and pandemics in humans are often caused by pathogens transmitted from non-human animal reservoirs. Influenza A viruses (IAVs) spread among a variety of different hosts and cross species barriers to create new viral strains. Waterfowl serves as the primary reservoir and can perpetuate many avian influenza virus (AIV) subtypes via asymptomatic shedding, which plays an important role in the reassortment and transmission of influenza subtypes to domestic poultry [1]. In 1996, a highly pathogenic (HP) AIV (H5N1) of Goose/Guangdong/1/96 (Gs/GD) lineage emerged in Chinese poultry and has been able to cross the species barrier and infect humans, which eventually spread to Europe, Africa, and the North American continent via migratory birds [2,3,4]. Due to the accumulation of genetic mutations and reassortment with multiple influenza subtypes, Gs/Gd lineage viruses evolved into nine clades and multiple subclades.

The phylogenetic clade 2.3.4.4 of H5Nx viruses has caused extensive outbreaks across the globe and has further evolved into eight subclades (2.3.4.4a–2.3.4.4h) [5]. In 2020/2021, clade 2.3.4.4b H5N1 viruses have spread to many countries in Europe, Africa, Asia, and America [6], and several infections have been reported in wild or captive mammals as well as in humans.

Highly pathogenic AIV (H5N1) was initially introduced into Egypt in 2005 and became endemic in poultry in 2008. Since then, many outbreaks have been reported in domestic poultry farms. Multiple clades of H5N1 Gs/Gd lineage viruses (Clades 2.2, 2.2.1, 2.2.1.1, 2.2.1.1a, and 2.2.1.2) were identified [7]. The 2.3.4.4b H5N8 virus was first detected in Egypt in wild birds in 2016 [8]. Since then, several cases of H5N8 were recorded among domestic poultry in live bird markets, backyard flocks, and commercial farms in several governorates in Egypt. Although all Egyptian H5N8 isolates belong to the clade 2.3.4.4, several independent introductions of the virus have been detected [9]. The 2.3.4.4b H5N8 replaced the clade 2.2.1 H5N1 viruses that subsequently disappeared. H9N2 AIVs were widespread in poultry globally and endemic in poultry in many Middle Eastern countries including Egypt, where H9N2 G1-like lineages were introduced in 2010 [10,11,12]. Extensive surveillance of the H9N2 virus has indicated that the virus was endemic in Egyptian domestic poultry in different geographical regions across the country and reassortant H9N2 viruses were detected [13]. Co-circulation of H5Nx and H9N2 viruses increases the probability of genetic reassortment which might enhance the zoonotic potential.

To monitor the influenza viruses with pandemic potential at the human–animal interface, in this study, we identify the genetic and antigenic characteristics of HPAI H5N1 viruses that were introduced into Egypt through active surveillance of AIVs in live bird markets (LBMs) and migratory wild birds.

## 2. Materials and Methods

### 2.1. Sample Collection

Active surveillance of avian influenza viruses has been conducted in Egypt through collaborative efforts of the Center of Scientific Excellence for Influenza viruses, National Research Centre, Egypt, and Center of Excellence for Influenza Research and Surveillance at St. Jude (Memphis, TN, USA) since 2009. In April 2021, we sampled poultry and wild birds sold in LBMs in Egypt. We collected cloacal and oropharyngeal swab samples, which were kept chilled in virus transport medium until they reached the laboratory.

### 2.2. Sample Screening and Virus Isolation

Samples were thoroughly vortexed prior to viral nucleic acid extraction from 200 μL of viral transport media using either the automated MagNA Pure 96 platform, KingFisher Flex instrument (Thermo Fisher Scientific, Rocklin, CA, USA) or the QIAamp Viral RNA Mini kit (Qiagen, Hilden, Germany), according to the manufacturer’s instructions. Nucleic acid extracts were screened by real-time RT-PCR (rRT-PCR) for the presence of the AIVs (universal M-gene) [14], and samples were classified as positive with a Ct value ≤ 36. All positive samples were individually injected into the allantoic cavity of 10-day-old specific pathogen-free embryonated hens’ eggs, incubated for 48 h post-injection at 37 °C, and then chilled at 4 °C for 4 h or overnight. Allantoic fluids were then collected and analyzed by the hemagglutination assay (HA) using 0.5% chicken red blood cells (RBCs). Hemagglutination assays (HA) of the allantoic fluids from the inoculated eggs were performed to screen for IAV according to the World Health Organization (WHO) and the World Organization for Animal Health (OIE) protocols. The positive samples were aliquoted and stored at −80 °C.

### 2.3. Sequencing and Sequence Analysis

Viral RNA extracted from allantoic fluid was subjected to reverse transcription to synthesize the first cDNA strand using a SuperScript IV first-strand synthesis kit (Invitrogen, Waltham, MA, USA) and the Uni12 influenza primer. Then, Phusion high-fidelity DNA polymerase (New England Biolabs, Ipswich, MA, USA) and Uni12/13 primers were used for multiplex PCR of all eight gene segments, and PCR products were purified. Sequencing library preparation was performed by using Illumina’s Nextera XT DNA Sample Preparation Kit according to the manufacturer’s protocol. Amplicons were sequenced on Illumina’s MiSeq platform (Illumina, San Diego, CA, USA) by using the paired-end approach. The eight full segments of each H5N8 virus were assembled using CLC Genomics Workbench, version 21 (CLC Bio, Qiagen, Hilden, Germany).

For sequence and phylogenetic analyses, genome sequences of H5Nx were aligned by MAFFT v4.787 [15], and the maximum likelihood (ML) trees were built from each segment alignment by FastTree v2.1.11 with GTR+Gamma model [16]. Temporal phylogeny was constructed by BEAST v1.10.4 under SRD06 substitution model [17,18], uncorrelated lognormal relaxed clock model, and Gaussian Markov random field (GMRF) Bayesian Skyride tree prior [19]. Two independent MCMC chains were run for 100 million iterations and sampled every 10,000 generations. Convergence was examined by Tracer v1.7.2 [20], requiring effective sample size (ESS) of over 200. The maximum clade credibility (MCC) tree was summarized by TreeAnnotator included in the BEAST package.

### 2.4. Antigenic Analysis

Haemagglutination inhibition (HI) assays were used to antigenically characterize the isolated viruses. The H5N1 AIVs were tested by using post-infection ferret antiserum raised against F.2015-7-A/duck/England/36254/2014 (H5N8), F.2017-13-A/chicken/Kumamoto/1-7/14 (H5N8), F.2016-16- A/gyrfalcon/Washington/410886/2014 (H5N8), and F.2015-48-A/Sichuan/26221/2014 (H5N6) of clade 2.3.4.4 viruses which were produced in Center of Excellence for Influenza Research and Surveillance at St. Jude (Memphis, TN, USA). A panel of post-infection ferret antisera was treated with receptor-destroying enzyme II and heat-inactivated at 56 °C for 30 min and diluted to a final concentration of 1:10 in PBS and 0.5% chicken erythrocytes. The HI test was performed according to the WHO protocols [21].

### 2.5. Nucleotide Sequence Accession Numbers

The nucleotide sequences of the H5N1 AIVs described in this study were deposited in the GenBank database with the accession numbers shown in Table S1.

## 3. Results and Discussion

Through surveillance, we isolated H5N1 viruses from one wild pintail duck and three domestic Pekin ducks, A/pintail/Egypt/RA19853OP/2021 in late 2021 and A/duck/Egypt/BA20360C/2022, A/duck/Egypt/BA20360OP/2022, and A/duck/Egypt/BA20361OP/2022 isolates in early 2022. The analysis of the complete HA gene segment showed that the HPAI H5N1 viruses belonged to phylogenetic clade 2.3.4.4b. The nucleotide sequence identities across all eight segments of the four viruses were 99.5–100%. As a representative virus, A/pintail/Egypt/RA19853OP/2021 (H5N1) had a high nucleotide identity (99–100%) to the HPAI A(H5N1) viruses of clade 2.3.4.4 from Europe and the Middle East (Table 1). These isolates were identified as HPAI viruses that harbored multiple basic amino acids (PLRERRRKR/G) within the cleavage site of the HA gene, which is characteristic of high pathogenicity in chickens.

**Figure 1:**
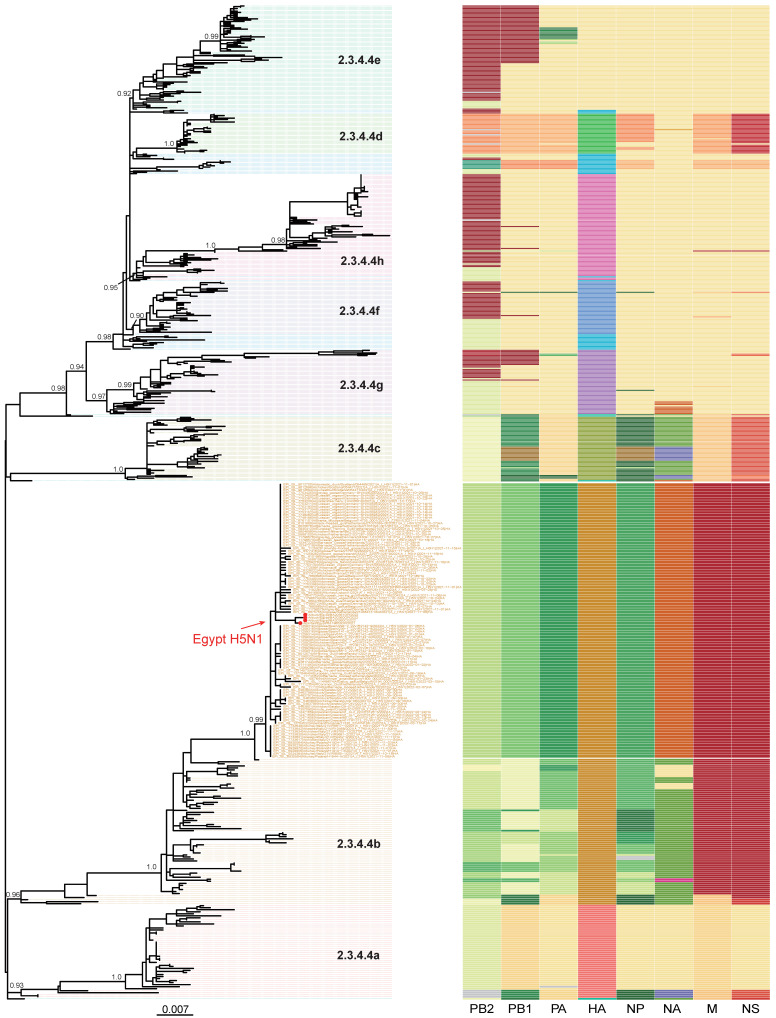
Phylogenetic tree of H5N1 viruses sequenced in this study, in addition to other publicly available H5Nx clade 2.3.4.4 from GenBank and GISAID. Red dots represent the H5N1 viruses sequenced in this study. Topological support values (SH-like support) of selected nodes are displayed. To the right, a schematic representation of viral clustering of each gene segment (from left to right: PB2, PB1, polymerase acidic, haemagglutinin, nucleoprotein, neuraminidase, matrix, and non-structural) is shown. Segment colors indicate origin of the segment. Within each cluster, a unified color pattern indicates homogeneity and a different color pattern indicates reassortment.

We combined genome sequences generated in this study with all sequences of H5Nx viruses available in GenBank and the GISAID database (11). Phylogenetic analysis confirmed that the Egyptian A(H5N1) isolates are of clade 2.3.4.4b and clustered with the recent HPAIV A(H5N1) isolates from Europe, Africa, and the Middle East (Figure 1). The clade 2.3.4.4b HA genes of H5 viruses have evolved from a sub-linage under clade 2.3.4.4 which includes several subtypes of H5N1, H5N6, and H5N8 viruses. Our isolates detected in this study clustered with the HA genes of H5N1 viruses contemporarily detected in Europe and the Middle East.

**Figure 2:**
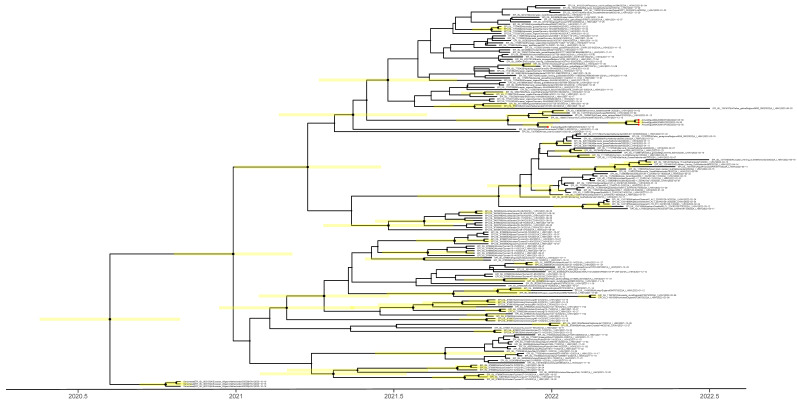
Time to the most recent common ancestor of H5N1 viruses sequenced in this study; maximum clade credibility temporal phylogeny of the hemagglutinin (HA) gene. The H5N1 viruses from Egypt are represented by red dots. Posterior clade probabilities are indicated by the sizes of the internal node circles. Shaded bars represent the 95% highest probability distribution for the age of each node with posterior clade probability > 0.3.

The time to the most recent common ancestor (tMRCA) was calculated to explain the emergence of the H5N1 viruses. Taking the intersection of the 95% highest posterior density (HPD) intervals of the tMRCA (Figure 2) suggests that the viruses from Europe, Africa, and the Middle East share a common ancestor of unknown origin that emerged around July 2020 (95%HPD: April 2020–October 2020). We did not find an amino acid deletion at position 133 in the HA protein (H3 numbering) in all our isolates, a feature common with clade 2.3.4.4 isolated from humans (Table 2), and associated with the alteration of the H5 HA receptor binding pocket [22]. The analysis of the NA gene of H5N1 viruses revealed that none of these viruses displayed oseltamivir resistance markers E119, H275, R293, and N295 (N1 numbering) (Table 2). Deletions were also present in both neuraminidase (NA) (an 11-aa deletion in the stalk region) and nonstructural protein 1 (NS1) (deletion from residues 80–84; Table 2), which are associated with high pathogenicity in avian hosts [23]. These analyses suggest that the newly detected H5N1 viruses in Egypt may be able to infect and cause disease in mammals.

The receptor binding sites in the viral HA gene of the four viruses possess the conservative amino acid residues (including 190E, 220R, 225G, 226Q, and 228G; H3 numbering), which indicated that these viruses would preferentially bind to the α-2,3-sialic acid linkage, the avian-like receptors.

The antigenic properties of H5N1 viruses were also assessed using ferret antisera against the World Health Organization’s candidate clade 2.3.4.4c H5N8 and clade 2.3.4.4a H5N6 vaccine viruses including A/gyrfalcon/Washington/41088-6/2014 (H5N8), A/duck/England/36254/2014 (H5N8), A/chicken/Japanese Kumamoto/1-7/2014 (H5N8), and A/Sichuan/26221/2014 (H5N6) (Table 3). The presence of E and D at positions 627 and 701 in polymerase basic (PB) 2 in viruses sequenced in this study also confirms a typical characteristic of avian influenza viruses (Table 2). PB2 amino acid substitutions L89V, E249G, G309D, and T339M enhance the replication and increased virulence of the H5N1 virus in mice [26,44], and the substitution L89V, G309D, and T339K were found in all isolates of our Egypt H5N1 viruses. PB1-F1 has been shown to contribute to viral pathogenicity, as well as to enhance inflammation, cytotoxicity, and viral polymerase activity [42,45]. All Egypt H5N1 isolates in this study expressed PB1-F2 of 90 aa and had the N66S mutation, which increases virulence, replication efficiency, and antiviral response in mice.

## 4. Conclusions

Several introductions of clade 2.3.4.4b viruses have been seen in Egypt. Those introductions are typically through wild migratory birds but eventually spill over to poultry. Some live bird markets in Northern Egypt sell both poultry and trapped wild birds for human consumption. Such an interface provides ample opportunity for cross-species virus spill-over. The viruses we detected were from such markets where the initial virus was detected in a migratory bird and then in domestic poultry. No human cases of clade 2.3.4.4b H5Nx infections were reported in Egypt but the mutations detected in analyzed viruses suggest that human infections can occur. A vigilant surveillance system at the human-wild bird-poultry interface is necessary.

## Figures and Tables

**Table 1:** Comparison of nucleotide sequence identities of the eight influenza A virus (IAV) gene sequences for the virus isolated in this study (A/pintail/Egypt/RA19853OP/2021 (H5N1)) and nearest virus homologs.

Gene *	Accession No.	Virus	Collection Date	% Identity
PB2	EPI2082687	A/Great white pelican/Israel/123/2022 (A/H5N1)	2022-01-12	99
	EPI1922962 †	A/duck/Saratov/29-08V/2021 (H5N1)	2021-09-30	99
PB1	EPI2085772	A/common kestel/Israel/49-2/2022 (A/H5N1)	2022-01-02	99.63
	EPI1922971	A/duck/Saratov/29-11V/2021 (A/H5N1)	2021-09-30	99.96
PA	EPI207623	A/chicken/Israel/88/2022 (A/H5N1)	2022-01-09	99
	EPI1922969	A/duck/Saratov/29-11V/2021 (A/H5N1)	2021-09-30	99
HA	EPI_ISL_418175	A/European herring gull/Sweden/SVA211116SZ0432/FB004518/M-2021 (A/H5N1)	2021-11-08	99
NP	EPI2085770	A/common kestel/Israel/49-2/2022 (A/H5N1)	2022-01-02	99
	EPI1922966	A/duck/Saratov/29-11V/2021 (A/H5N1)	2021-09-30	99
NA	EPI2085768	A/common kestel/Israel/49-2/2022 (A/H5N1)	2022-01-02	99
	EPI1963383	A/guineafowl/Scotland/054471/2021 (A/H5N1)	2021-11-01	99
M	EPI2085769	A/common kestel/Israel/49-2/2022 (A/H5N1)	2022-01-02	100
	EPI1922960	A/duck/Saratov/29-08V/2021 (A/H5N1)	2021-09-30	100
NS	EPI2008127	A/black-headed_gull/England/388256/2022 (A/H5N1)	2022-02-03	99
	EPI1938848	A/barnacle goose/Sweden/SVA211111SZ0376/FB004496/2021 (A/H5N1)	2021-11-01	99

* PB2, basic polymerase 2; PB1, basic polymerase 1; PA, acidic polymerase; HA, hemagglutinin; NP, nucleoprotein; NA, neuraminidase; MP, matrix protein; NS, nonstructural protein. † Nearest virus homologs to A/pintail/Egypt/RA19853OP/2021 (H5N1) isolated before of detection time.

**Table 2:** Assessment of molecular amino acid markers for zoonotic potential of the influenza A(H5N1) virus detected in Egypt.

Viral Protein	Amino Acid	A/Pintail/Egypt/RA19853OP/2021 (H5N1)	Functional Relevance	References
PB2	E627K	E	Mammalian host adaptation	[24]
	D701N	D	Increase polymerase activity and viral replication in mammalian cells	[25]
	L89V	V	Enhanced polymerase activity, increased virulence in mice	[26]
	G309D	D		
	T339K	K		
	A588V	A	Mammalian host adaptation	[27]
PB1-F2	N66S	S	Increases virulence, replication efficiency, and the antiviral response in mammals	[28,29]
PA	V100A	V	Contributed to the virulence and mammalian adaptation	[30]
	S409N	S		
	A515T	T	Increased polymerase activity, increased virulence in mammals and birds	[31]
HA	E198D	E	Enhanced mammalian receptor binding	[32]
	Q234L	Q	Preferential binding to human Sialic acid α2–6 receptor	[33,34]
	G236S	G		
	I155T	T	Enhanced mammalian receptor binding	[35]
NA	E119V	E	Oseltamivir resistance	[36,37]
	H275Y	H		
	R293K	R		
	N295S	N		
M2	L26P	L	Reduced susceptibility to amantadine	[38,39,40]
	V27A/I	V		
	A30T	A		
	S31N	S		
	G34E	G		
NS1	P42S	S	Increased virulence and pathogenicity in mammals	[41,42,43]
	D92E	D		
	V149A	A		

**Table 3:** Antigenic analysis of H5N1 influenza A viruses from Egypt by hemagglutination inhibition assay.

	F.2015-7 *	F.2017-13	F.2016-16	F.2015-48
	A/duck/England/36254/2014	A/chicken/Kumamoto/1-7/14	A/Gyrfalcon/WA/41088/2014	A/Sichuan/26221/2014
A/duck/England/36254/2014	**320**	2560	1280	2560
A/chicken/Kumamoto/1-7/14	40	**80**	<40	<40
A/Gyrfalcon/WA/41088/2014	160	640	**320**	320
A/Sichuan/26221/2014	80	80	<40	**80**
A/Pintail/Egypt/RA19853OP/2021	320	640	640	40
A/duck/Egypt/BA20360C/2022	160	640	320	160
A/duck/Egypt/BA20361C/2022	640	1280	1280	1280
A/duck/Egypt/BA20361OP/2022	160	640	320	160

* Polyclonal antibodies were produced in ferrets. Homologous titers are bold and underlined.

## Data Availability

All data used in this study are available on request from the corresponding author.

## Supplementary Materials

The following supporting information can be downloaded at: https://www.mdpi.com/article/10.3390/pathogens12010036/s1, Table S1: GenBank Accession Number.
