# Correction: El-Shesheny et al. Highly Pathogenic Avian Influenza A(H5N1) Virus Clade 2.3.4.4b in Wild Birds and Live Bird Markets, Egypt. *Pathogens* 2023, *12*, 36

**DOI:** 10.3390/pathogens13090726

**Published:** 2024-08-27

**Authors:** Rabeh El-Shesheny, Yassmin Moatasim, Sara H. Mahmoud, Yi Song, Ahmed El Taweel, Mokhtar Gomaa, Mina Nabil Kamel, Mohamed El Sayes, Ahmed Kandeil, Tommy T. Y. Lam, Pamela P. McKenzie, Richard J. Webby, Ghazi Kayali, Mohamed Ahmed Ali

**Affiliations:** 1 Center of Scientific Excellence for Influenza Viruses, National Research Centre, Giza 12622, Egypt; 2 State Key Laboratory of Emerging Infectious Diseases, School of Public Health, The University of Hong Kong, Hong Kong SAR, China; 3 Laboratory of Data Discovery for Health Limited, Hong Kong SAR, China; 4 Department of Infectious Diseases, St. Jude Children’s Research Hospital, Memphis, TN 38105, USA; 5 Centre for Immunology & Infection Limited, Hong Kong SAR, China; 6 Human Link, Dubai 3O-01-BA380, United Arab Emirates

**Text Correction**

In the original publication [1], there was an error in the third sentence of the third paragraph in Section 3. The sentence “We also found an amino acid deletion at position 133 in the HA protein (H3 numbering) in all our isolates” should be changed to “We did not find an amino acid deletion at position 133 in the HA protein (H3 numbering) in all our isolates”. 

The authors state that the scientific conclusions are unaffected. This correction was approved by the Academic Editor. The original publication has also been updated.
