# Supplementary material for: Highly Pathogenic Avian Influenza A(H5N1) Virus Clade 2.3.4.4b in Wild Birds and Live Bird Markets, Egypt
Source: Pathogens. 2022 Dec 26;12(1):36. doi: 10.3390/pathogens12010036 (PMC9866256; doi:10.3390/pathogens12010036)
Supplement: Supplementary file 1 [file pathogens-12-00036-s001.zip › pathogens-2062895-supplementary.pdf]

Table S1: GenBank Accession Number

Segment	A/duck/Egypt/BA20360C/2022	A/pintail/Egypt/RA19853OP/2021	A/duck/Egypt/BA20361OP/2022	A/duck/Egypt/BA20360OP/2022
PB2	OP590394	OP590530	OP590514	OP590410
PB1	OP590395	OP590531	OP590515	OP590411
PA	OP590396	OP590532	OP590516	OP590412
HA	OP590397	OP590533	OP590517	OP590413
NP	OP590398	OP590534	OP590518	OP590414
NA	OP590399	OP590535	OP590519	OP590415
MP	OP590400	OP590536	OP590520	OP590416
NS	OP590401	OP590537	OP590521	OP590417
